# Cross-species transmission of *bla*_NDM-5_-IncX3 plasmids: molecular mechanisms and One Health-based strategies for CREC control

**DOI:** 10.3389/fcimb.2026.1783836

**Published:** 2026-04-13

**Authors:** Benjin Xu, Ling Liu, Hui Fu, Meicheng Liu, Xiaoyan Li

**Affiliations:** 1 Department of Medical Laboratory Science, Shanxi University of Medicine, Fenyang, China; 2 Graduate School, Shanxi Medical University, Taiyuan, China; 3 Department of Clinical Laboratory, Fenyang Hospital of Shanxi Province, Fenyang, China; 4 Science and Technology Centre, Shanxi University of Medicine, Fenyang, China

**Keywords:** *bla*_NDM-5_-IncX3, CREC, molecular epidemiology, One Health, transmission mechanism

## Abstract

Carbapenem-resistant *Escherichia coli* (CREC) has emerged as a critical global health concern, particularly strains carrying the *bla*_NDM-5_-IncX3 plasmid. In this review, we synthesize recent advances in the molecular epidemiology, resistance mechanisms especially the transmission dynamics of the *bla*_NDM-5_-IncX3 plasmid, virulence factors, research methodologies, and current strategies for the prevention and control of CREC. We further explore the cross-species dissemination and adaptive evolution of *bla*_NDM-5_-IncX3 plasmids within the interconnected human-animal-environment interface. Based on these insights, we propose a novel triadic transmission model—”structural adaptation, host reciprocity, and environmental driving”, alongside a One Health-based, multidimensional intervention framework spanning clinical, agricultural, and environmental domains. This review provides critical perspectives for the containment of carbapenem-resistant *Enterobacteriaceae*, offers theoretical support for anti-infective clinical practice, informs evidence-based public health policy, and facilitates the implementation of the One Health approach in antimicrobial resistance (AMR) governance.

## 1 Introduction

*Escherichia coli* (*E. coli*) is a prevalent opportunistic pathogen causing both hospital- and community-acquired infections, including gastroenteritis, urinary tract infections, and septicemia (Kaper et al., 2004; Croxen and Finlay, 2010; Teng et al., 2023). In recent years, the increasing prevalence of extended-spectrum β-lactamase (ESBL)-producing strains has driven resistance to β-lactam antibiotics, rendering carbapenems the last-resort treatment for severe Gram-negative bacterial infections (Harris et al., 2015; Laxminarayan et al., 2016; Kizny Gordon et al., 2020; Moon et al., 2023; Yuan et al., 2024). However, the expanding clinical use of carbapenems has been accompanied by a steady rise in carbapenem-resistant *E. coli* (CREC), posing major challenges to antimicrobial therapy and hospital infection control (Bonomo et al., 2018; Sabour et al., 2021; Xu et al., 2023; Tamma et al., 2024). Alarmingly, although carbapenems are not approved for veterinary use, CREC has been widely detected in animal husbandry, with evidence of transmission between animals and humans via food chains and environmental pathways, constituting a serious public health threat (Wang et al., 2017; Shen et al., 2018; Wu et al., 2019; Hong et al., 2020; Liu et al., 2023). Consequently, the World Health Organization has designated carbapenem-resistant Enterobacterales as critical priority pathogens for novel drug development (WHO, 2024; Macesic et al., 2025).

**Figure 1:**
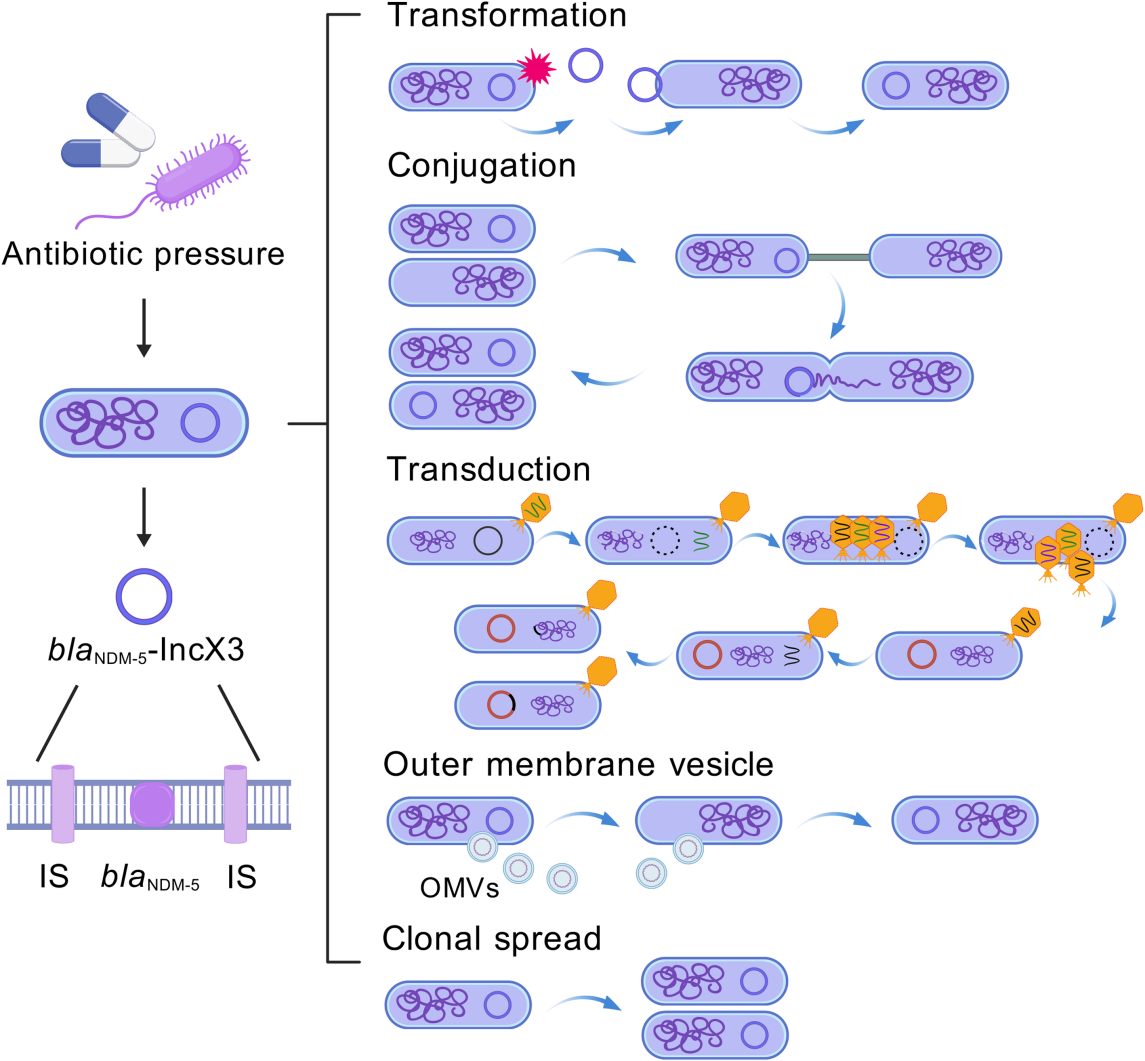
Main transmission mechanisms of *bla*_NDM-5_-IncX3 plasmids. This image illustrates the major transmission mechanisms of the *bla*_NDM-5_-IncX3 plasmid carried by CREC under antibiotic selection pressure. The carbapenemase gene *bla*_NDM-5_, flanked on both sides by insertion sequences, is located within the variable region of the IncX3 plasmid. The dissemination of this resistance plasmid is a major driver of large-scale CREC epidemics. Transformation, conjugation, transduction, and outer membrane vesicles (OMVs) (Fulsundar et al., 2014; Johnston et al., 2014; Tashiro et al., 2017; Tran and Boedicker, 2017; Toyofuku et al., 2018; Arnold et al., 2021; Toyofuku et al., 2023) constitute the primary routes of horizontal gene transfer (HGT). Transformation depends on the presence of intact, undegraded plasmid molecules in the environment, and plasmid–host compatibility influences transfer efficiency. Conjugation is the most efficient and specific route of dissemination, with the presence of conjugative plasmids being critical to this process. Transduction is mediated by bacteriophages, which during infection can carry plasmid fragments (including antibiotic resistance genes, shown in black) from donor bacteria and transfer them to recipient bacteria. The release and uptake of OMVs represent an important mechanism of HGT within biofilm environments. Clonal spread is a key mode of vertical transmission for resistance plasmids. Graphical elements in this figure are adapted from the Generic Diagramming Platform (GDP) (Jiang et al., 2025).

The rapid dissemination of CREC is primarily driven by horizontal transfer of antibiotic resistance genes (ARGs) mediated by mobile genetic elements (MGEs), including plasmids, insertion sequences, and transposons (Evans et al., 2020; Zhang et al., 2021; Xiang et al., 2024). ARGs spread through conjugation, transduction, and transformation, with conjugation representing the dominant mechanism (**Figure 1**) (Wright, 2007; Johnston et al., 2014; Ravi et al., 2018; Jiang et al., 2020; Lian et al., 2023; Xiang et al., 2024). A key contributor to CREC emergence is New Delhi metallo-β-lactamase (NDM), encoded by *bla*_NDM_, which is frequently carried on IncX3 plasmids characterized by high transfer efficiency and low fitness cost (Johnson et al., 2012; Khabbaz et al., 2014; Wu et al., 2019; Macesic et al., 2024; Yang et al., 2024). The global emergence of CREC, particularly strains harboring *bla*_NDM-5_-IncX3 plasmids, has raised substantial concern due to limited therapeutic options, increased treatment complexity, and elevated mortality risk. Therefore, elucidating the molecular epidemiology of CREC and the transmission dynamics of *bla*_NDM-5_-IncX3 plasmids is essential for improving infection control and developing effective prevention strategies. To maintain a CREC-centered perspective, the following sections examine *bla*_NDM-5_ and IncX3 plasmids within the broader context of CREC dissemination, evolution, and control.

## 2 CREC: a rising global threat with complex transmission dynamics

Antimicrobial resistance (AMR) represents a major global health challenge within the One Health framework, undermining the prevention and treatment of infections in both humans and animals (Wright, 2007; Laxminarayan et al., 2016; Hickman et al., 2021; Coque et al., 2023; GBD 2021 Antimicrobial Resistance Collaborators, 2024; Okeke et al., 2024; Macesic et al., 2025; Theuretzbacher, 2025; Theuretzbacher et al., 2025). Carbapenems are regarded as the last-resort therapy for severe infections caused by multidrug-resistant Gram-negative bacteria (Harris et al., 2015; Kizny Gordon et al., 2020; Moon et al., 2023; Yuan et al., 2024), yet carbapenem resistance has increasingly been reported in *E. coli* over the past two decades (Li et al., 2021; Ba et al., 2024). Surveillance data from China and the United States demonstrate a sustained increase in CREC prevalence, with CREC accounting for up to 2.5% of *E. coli* isolates in the US (CDC, 2022; Wilson et al., 2017; Zhao et al., 2021; Miller and Arias, 2024). CREC commonly exhibits resistance to multiple antibiotic classes, including carbapenems (e.g., imipenem and meropenem) and extended-spectrum cephalosporins, complicating clinical management and threatening global health security. Notably, many CREC isolates do not produce carbapenemases; instead, resistance is frequently mediated by porin loss combined with amplification of ESBL gene copy numbers (Logan and Weinstein, 2017; van Duin et al., 2020). The diversity of resistance mechanisms underscores the complexity of CREC epidemiology and complicates hospital infection prevention efforts (Miller and Arias, 2024). Recent studies further confirm CREC as a major pathogen associated with nosocomial infections worldwide (Peirano et al., 2022; Ding et al., 2023).

CREC populations comprise diverse sequence types (STs) with distinct geographic distributions. A multicenter study across 36 countries identified ST410, ST131, ST167, and ST405 as predominant global lineages (Peirano et al., 2022; Zhang et al., 2023), while surveillance in China revealed ST167 and ST131 as dominant clones, with ST410 also prevalent (Zhang et al., 2017; Li et al., 2021; Wang et al., 2025). CREC transmission occurs via contaminated medical equipment, healthcare worker contact, patient-to-patient spread, and environmental reservoirs (Allen et al., 2010; Ding et al., 2023; Zhang et al., 2023). Beyond healthcare settings, CREC has been detected in communities, animals, food products, and wastewater, highlighting its capacity for cross-sector dissemination (Allen et al., 2010; Holmes et al., 2016; Wang et al., 2023; Zhang et al., 2023; Li et al., 2024b). Resistance genes such as *bla*_NDM-5_, *bla*_KPC-2_, and *bla*_OXA-48_ exhibit interregional spread, and shared *bla*_NDM-5_-IncX3 plasmids between human and animal isolates indicate cross-species transmission potential (Zhang et al., 2023; Li et al., 2024b).

The continued emergence of CREC emphasizes the need for strengthened global surveillance, molecular epidemiological investigation, and coordinated control strategies. High-resolution approaches such as whole-genome sequencing, multi-locus sequence typing, and single nucleotide polymorphism analysis enable tracing of transmission routes, identification of clonal lineages, and characterization of resistance mechanisms (Tenaillon et al., 2010; Ladner et al., 2019; Jamin et al., 2021; Suleyman et al., 2024; Wang et al., 2025), providing a critical foundation for effective CREC prevention and control.

## 3 *bla*_NDM-5_: a key genetic determinant mediating carbapenem resistance

CREC emergence is largely driven by acquisition of carbapenemase-encoding genes capable of hydrolyzing carbapenems and other β-lactams (Nordmann and Poirel, 2019; Antimicrobial Resistance Collaborators, 2022; Macesic et al., 2025). Carbapenemases are classified into serine β-lactamases (e.g., KPC, IMI, OXA-48-like) and metallo-β-lactamases (MBLs), including NDM, VIM, and IMP, which require zinc ions for activity (Bush and Bradford, 2020; Li et al., 2021; Zhang et al., 2025). These genes are typically embedded in MGEs such as plasmids, transposons, and insertion sequences, facilitating horizontal dissemination across bacterial species (Wu et al., 2019; Zhang et al., 2021).

NDM is among the most prevalent carbapenemase detected in *E. coli*, conferring resistance to nearly all β-lactam antibiotics and posing a severe public health threat (Cole et al., 2020; Marchetti et al., 2020). Since its initial identification in 2008, *bla*_NDM_ has disseminated globally, with more than 40 variants reported worldwide (Yong et al., 2009; Naas et al., 2017; Pál et al., 2017; Lu et al., 2020; Zeng et al., 2022). Among these, *bla*_NDM-5_, first identified in *E. coli* in 2011 (Hornsey et al., 2011), has become one of the most widespread variants (Baek et al., 2019; Lu et al., 2020; Tian et al., 2020). It is carried on diverse plasmid backbones, including IncX3, IncHI2, and IncF, which play key roles in its epidemiology (Turton et al., 2022; Zeng et al., 2023).

NDM-5 differs from NDM-1 by two amino acid substitutions (V88L and M154L), which have been shown in biochemical and comparative studies to enhance hydrolytic activity against carbapenems, cefotaxime, and ceftazidime, resulting in higher resistance levels (Li et al., 2024). *bla*_NDM-5_ frequently coexists with other resistance genes, such as *bla*_CTX-M_, *bla*_OXA-181_, and *bla*_OXA-48_, forming multidrug resistance clusters that severely restrict therapeutic options (Di Marcantonio et al., 2024; Zuo et al., 2024). Some isolates additionally harbor resistance determinants against colistin, tigecycline, or aminoglycosides, further compounding treatment challenges (Sun et al., 2016; Ho et al., 2018; Long et al., 2019; Li et al., 2023).

Epidemiological studies identify ST48, ST167, and ST10 as major *bla*_NDM-5-_ associated *E. coli* lineages in humans (Liu et al., 2019; Sun et al., 2019; Wang et al., 2021). The detection of NDM-5-producing *E. coli* in pets, food-producing animals, and environmental samples highlights their role as reservoirs for community dissemination (Almakki et al., 2017; Bleichenbacher et al., 2020; Hooban et al., 2020; Li et al., 2021; Zhao et al., 2021; Tsilipounidaki et al., 2022; Ma et al., 2024; Moon et al., 2024). Nevertheless, the transmission dynamics of *bla*_NDM-5_ across the human-animal-environment interface remain incompletely understood.

## 4 The IncX3 plasmid: a conserved yet adaptable vehicle for *bla*_NDM-5_ dissemination

The *bla*_NDM-5_ gene has been identified on diverse plasmid backbones, including IncX3, IncF, IncN, and hybrid plasmids such as IncX3-IncFIB (Li et al., 2018; Wu et al., 2019; Zhai et al., 2020; Zou et al., 2020). Among 355 *bla*_NDM_-harboring plasmids deposited in GenBank, 117 (~33%) contain the IncX3 replicon (Wu et al., 2019). *bla*_NDM-5_-positive IncX3 plasmids have been widely detected in *Enterobacteriaceae* from human, animal, and environmental sources across multiple regions, including Asia, Africa, and Oceania (Krishnaraju et al., 2015; Wailan et al., 2015; Brinkac et al., 2019; Sekizuka et al., 2019; Kayama et al., 2022) (**Box 1**). Notably, 68.4% of IncX3 entries originate from East and Southeast Asia, particularly China, South Korea, Vietnam, and Myanmar, highlighting IncX3 plasmids as major vehicles for *bla*_NDM-5_ dissemination in this region (Wu et al., 2019).

Box 1 **Major bacterial hosts of *bla*_NDM-5_- IncX3 plasmids.**

*bla*_NDM-5_-bearing IncX3 plasmids have been detected in a broad range of Gram-negative bacteria from clinical, animal, and environmental sources. The following species represent key reservoirs contributing to the global dissemination of these plasmids:

**Table:** 

Bacterial species	Reservoirs	Notable features
*Escherichia coli*	Human, animal, environment	Primary host; multiple high-risk STs (e.g., ST167)
*Klebsiella pneumoniae*	Hospital, ICU	Frequently co-harbors other β- lactamases
*Citrobacter freundii*	Wastewater, soil	Environmental persistence and recombination potential
*Salmonella enterica*	Poultry, meat	Foodborne zoonotic risk
*Pseudomonas aeruginosa*	Healthcare, settings	Acquires plasmids via interspecies conjugation

The broad host range and cross-ecological presence of these species underscore the urgent need for One Health-based monitoring and intervention strategies.

IncX3 plasmids are typically small (~46 kb), highly transmissible, genetically stable, and capable of dissemination among diverse Gram-negative bacteria, including *E. coli*, *Klebsiella* spp., and *Salmonella* spp (Johnson et al., 2012; Ho et al., 2018; Shen et al., 2018). Comparative genomic analyses demonstrate a high degree of sequence conservation among *bla*_NDM-5_-carrying IncX3 plasmids isolated from different continents (González-Espinosa et al., 2024). Their backbone architecture is highly conserved and comprises core functional modules responsible for replication, partitioning, conjugative transfer, and regulation. Replication is controlled by the *repA-oriV* system, ensuring stable plasmid maintenance, while the *parA-parB* partitioning system promotes faithful segregation during cell division (Guo et al., 2022). RepA-mediated regulation of oriV also contributes to plasmid copy number control, and stress conditions may induce shifts in copy number, potentially influencing resistance gene expression and host adaptation. Conjugative transfer is mediated by a type IV secretion system (T4SS), encoded by *virB/virD4-*associated genes, which enables efficient intercellular plasmid transmission (Ilangovan et al., 2015; Costa et al., 2021; Macé et al., 2022; Li et al., 2024a).

Recent studies have identified regulatory proteins that modulate IncX3 conjugation efficiency in specific experimental systems. The NusG family protein PrfaH and transcriptional regulator VirBR have been shown to enhance T4SS gene expression and promote plasmid transfer in certain hosts or plasmid variants (Ma et al., 2024; Yang et al., 2025). In contrast, H-NS-like regulators may repress conjugation-related genes while contributing to bacterial persistence and host adaptation, indicating a finely tuned balance between plasmid dissemination and host fitness (Liu et al., 2020; Yang et al., 2025).

Based on genomic variation within resistance regions, IncX3 plasmids can be classified into at least nine subgroups, such as pNDM-HN380 and pNDM-MGR194 (Liu et al., 2021). While the plasmid backbone remains conserved, the resistance region is highly variable and frequently harbors multiple antimicrobial resistance genes, including *bla*_NDM-5_, *bla*_OXA-181_, *bla*_KPC_, and the colistin resistance gene *mcr-1* (Sun et al., 2016; Zuo et al., 2024). These genes are commonly embedded within transposons (e.g., *Tn125* and *Tn3* families) or integrons and flanked by insertion sequences such as *IS26*, *IS5*, and *ISAba125*, forming composite transposon structures (Mazel, 2006; Sun et al., 2016). In particular, IS26 plays a pivotal role in mediating recombination events, including the formation of hybrid plasmids co-harboring *bla*_NDM-5_ and *mcr-1*, thereby facilitating the simultaneous dissemination of resistance to carbapenems and colistin (Sun et al., 2016).

The evolution of IncX3 plasmids is closely linked to the acquisition and rearrangement of MGEs. In addition to resistance determinants, some IncX3 plasmids carry heavy metal resistance genes, which may enhance bacterial survival under environmental selective pressures (Ma et al., 2024). Importantly, IncX3 plasmids generally impose minimal fitness costs on their hosts, which may be attributed to their relatively small size, conserved backbone structure, and efficient replication and partitioning systems. These features reduce the metabolic burden on host cells and facilitate stable plasmid maintenance. IncX3 plasmids may even promote environmental adaptability, including biofilm formation, further supporting their persistence and spread. In addition, variation in plasmid copy number may influence *bla*_NDM-5_ expression levels and contribute to host fitness trade-offs, balancing enhanced resistance with the metabolic burden imposed on the host cell.

Overall, IncX3 plasmids combine a conserved backbone with a highly plastic resistance region, conferring both structural stability and genetic flexibility (**Box 2**). These features underpin their central role in the global dissemination of *bla*_NDM-5_. Elucidating the functional interplay between conjugation-associated genes, regulatory networks, and environmental pressures will be critical for understanding the transmission dynamics and host adaptation of *bla*_NDM-5_-IncX3 plasmids.

Box 2 **Structure and genetic organization of *bla*_NDM-5_-carrying IncX3 plasmids.**

IncX3 plasmids represent a compact yet highly efficient vehicle for the dissemination of *bla*_NDM-5_, with distinct genetic modules conferring both structural stability and adaptive versatility:

• Conserved backbone (~46 kb):

Replication system: *repA* and *oriV* ensure stable plasmid maintenance.
Partitioning system: *parA*/*parB* facilitate equal segregation.
Conjugation machinery: *virB*/*virD4* encode the T4SS.
Regulatory elements: H-NS-like proteins fine-tune expression of transfer and virulence genes.

• Variable resistance region:

Resistance determinants: *bla*_NDM-5_, *bla*_oxa-181_, *mcr-1*, *qnrS1*, and *rmtB*.
Mobile genetic elements: insertion sequences (IS26, ISAba125), transposons (Tn125), and integrons.
Composite transposons: e.g., IS3000-IS5-ISAba125-*bla*_NDM-5_-ble-trpF-dsbC-IS26.

This modular architecture enables IncX3 plasmids to retain high transmissibility and adaptability across diverse hosts, driving their success in the global spread of carbapenem resistance.

## 5 Cross-species dissemination of the *bla*_NDM_-5-IncX3 plasmid: molecular mechanisms and One Health drivers

The *bla*_NDM-5_-IncX3 plasmid disseminates through multiple routes, including conjugative transfer, transposon-mediated mobilization, plasmid co-residence and co-evolution, and environmental spread. Among these, conjugation is the primary mechanism driving interspecies transmission. IncX3 plasmids encode a complete set of conjugation-associated genes (*tra* genes), enabling efficient transfer among diverse bacterial hosts, including *E. coli*, *Klebsiella pneumoniae*, and *Pseudomonas aeruginosa* (Tian et al., 2020). This broad host range positions the *bla*_NDM-5_-IncX3 plasmid as a major vector for the global dissemination of carbapenem resistance.

Recent studies have identified VirBR as a transcriptional regulator that binds to the promoter region of *actX*, thereby enhancing expression of the type IV secretion system (T4SS) required for plasmid conjugation in certain experimental systems (Ma et al., 2024). In selected strains carrying *bla*_NDM-5_-IncX3 plasmids, VirBR-mediated activation of T4SS has been shown to increase plasmid transfer efficiency. Environmental metals such as Cu^2+^ and Zn^2+^ have also been reported to induce VirBR expression in these model systems, potentially promoting plasmid dissemination under environmental selective pressures (Berg et al., 2005; Seiler and Berendonk, 2012; Zhang et al., 2019; Raro et al., 2023). In addition, *bla*_NDM-5_ is embedded within the transposon *Tn125*, which carries *ISAba125* and facilitates recombination between plasmid and chromosomal DNA across bacterial species, thereby expanding the resistance gene reservoir (Sun et al., 2016).

Beyond conjugation, IncX3 plasmids can stably coexist with other resistance plasmids, including IncF and IncA/C, within the same host cell, resulting in multidrug-resistant phenotypes encompassing resistance to carbapenems, aminoglycosides, and fluoroquinolones (Tian et al., 2020). Toxin-antitoxin (TA) systems encoded by IncX3 plasmids further enhance plasmid stability and prevents loss during bacterial replication (Ni et al., 2021). Together, these features facilitate plasmid persistence and co-evolution within bacterial populations.

Outside clinical settings, CREC strains carrying *bla*_NDM-5_-IncX3 plasmids can disseminate through contaminated medical devices, direct patient contact, and inadequate hand hygiene (Ding et al., 2023; Zhang et al., 2023). Alarmingly, these plasmids have also been detected in food products and environmental matrices such as wastewater, indicating active circulation across the human-animal-environment interface (Wang et al., 2023). Under selective pressure from broad-spectrum antibiotics, particularly carbapenems and cephalosporins, CREC strains harboring *bla*_NDM-5_-IncX3 gain a competitive advantage, accelerating their expansion in high-antibiotic-use environments such as hospitals and long-term care facilities (Ma et al., 2024).

Collectively, the dissemination of *bla*_NDM-5_-IncX3 plasmids is driven by the interplay of efficient conjugation, genomic mobilization, cross-species transmission, and antimicrobial selection pressure (**Box 3**). Understanding these mechanisms is essential for developing strategies to curb the global spread of carbapenem resistance.

Box 3 **One Health reservoirs and transmission pathways of CREC.**

The spread of carbapenem-resistant *E. coli* (CREC) carrying *bla*_NDM-5_-IncX3 plasmids spans interconnected ecosystems. The following are key transmission nodes within the One Health framework:

• Human: Hospitals and long-term care facilities act as amplification hubs through nosocomial transmission, colonized patients, and inadequate hand hygiene. Community carriers also contribute to fecal dissemination.
• Animal: Antibiotic use in livestock promotes colonization. CREC can be transmitted through contaminated meat and slaughterhouse environments.
• Environment: Wastewater treatment plants (WWTPs), agricultural runoff, and surface waters serve as resistance gene reservoirs. Biofilms enhance CREC persistence.

These reservoirs form a “resistance gene eco-cycle”, wherein human, animal, and environmental compartments continuously exchange resistance determinants, reinforcing the importance of integrated surveillance and control.

## 6 Virulence factors of CREC and their role in pathogenicity

The evolution of virulence in *E. coli* is driven by three principal mechanisms: acquisition of new genes via horizontal gene transfer mediated by MGEs, inactivation of antivirulence genes, and pathoadaptive point mutations that remodel existing functions (Sokurenko et al., 1999; Bliven and Maurelli, 2012; Arnold et al., 2021; Denamur et al., 2021). A well-characterized example is the modification of the type I fimbrial adhesin FimH, which enhances host cell adhesion and colonization (Sokurenko et al., 1998).

**Figure 2:**
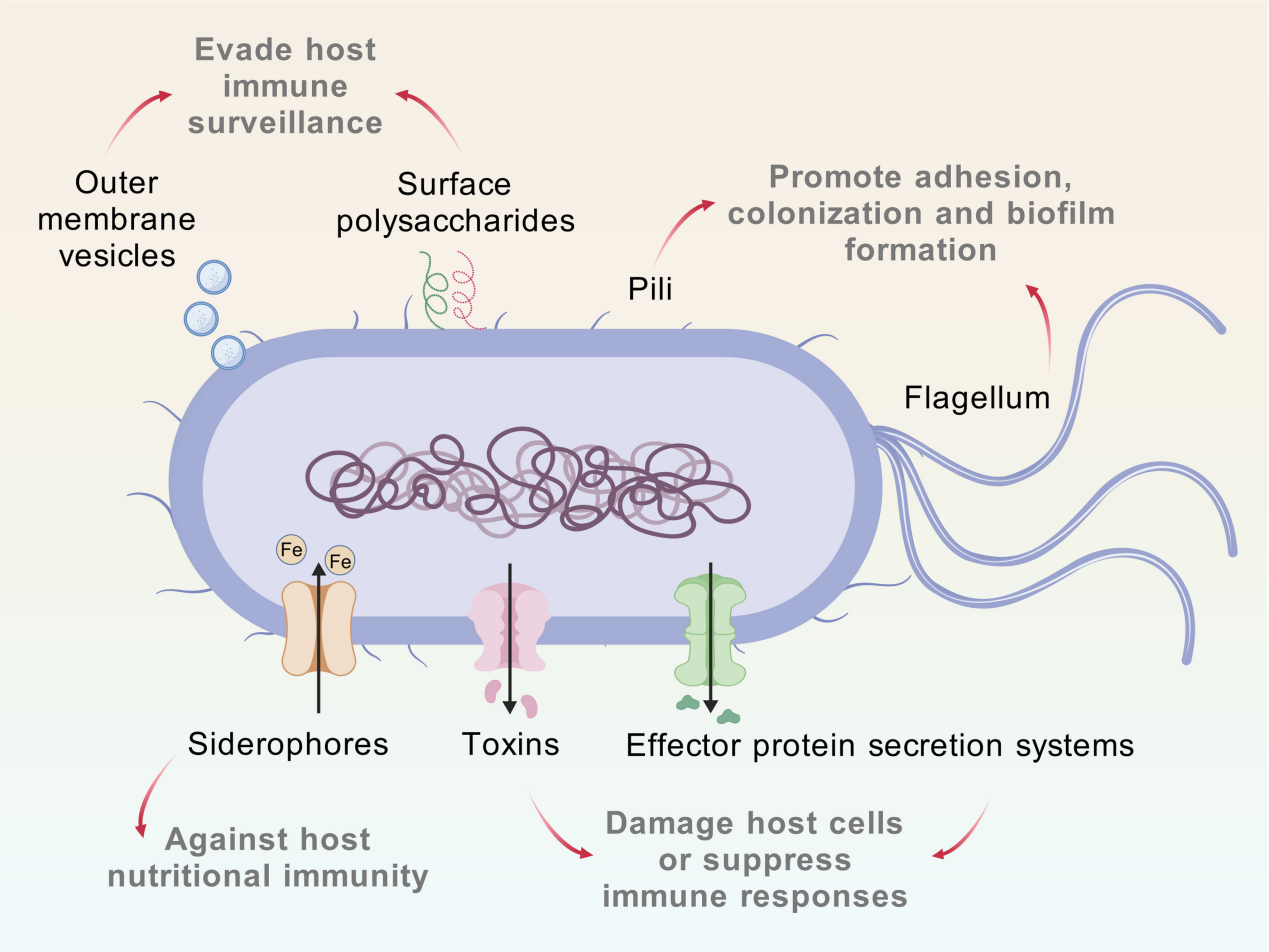
Key virulence factors of CREC and associated pathogenic mechanisms. This image summarizes the major virulence factors of CREC and their pathogenic mechanisms. The pathogenic process of CREC can be outlined as “adhesion and colonization – invasion/toxin-mediated damage – immune evasion/suppression,” with different types of virulence factors acting at each stage. Flagella and fimbriae are primarily responsible for bacterial adhesion and colonization at the site of infection, and also play important roles in quorum-sensing-related processes such as biofilm formation. Toxins and effector protein secretion systems are the main virulence factors that cause host cell damage, enhancing bacterial survival and suppressing host immune responses. Siderophores are key virulence factors that enable bacteria to resist host nutritional immunity; in addition to promoting bacterial colonization and invasion, they can also alter cell membrane permeability, linking bacterial virulence with antimicrobial resistance. Surface polysaccharides and outer membrane vesicles (Toyofuku et al., 2023) contribute to bacterial evasion of host immune surveillance and suppression of host immune responses. Graphical elements in this figure are adapted from the Generic Diagramming Platform (GDP) (Jiang et al., 2025).

The virulence of CREC is closely associated with the diversity and abundance of its virulence factors (**Figure 2**). Global surveillance indicates that clinical CREC isolates harbor more host-adaptive virulence determinants than isolates from healthy individuals or environmental sources (Huang et al., 2024). Among these, siderophores play a central role in pathogenicity. By secreting siderophores such as aerobactin, CREC efficiently scavenges iron under iron-limited conditions, promoting bacterial survival, colonization, and invasion. Aerobactin is enriched in hypervirulent lineages such as ST410, and its gene cluster (*iucABCD-iutA*) is frequently located on plasmids co-harboring *bla*_NDM-5_, forming mobile elements that couple multidrug resistance with enhanced virulence (Ba et al., 2024; Li et al., 2024b).

Virulence factors may also indirectly contribute to antimicrobial resistance by altering membrane permeability and reducing antibiotic penetration. Adhesins and pili, including FimH and type I fimbriae, facilitate attachment to epithelial surfaces and promote biofilm formation. Overexpression of *fimH* is strongly associated with urinary tract and catheter‐associated infections, where biofilm impedes antibiotic efficacy and enhances persistence (Ba et al., 2024). Biofilm-associated genes such as *csgA* are frequently detected in environmental CREC isolates, highlighting their role in nosocomial transmission and environmental survival (Li et al., 2024b).

Some CREC strains additionally produce exotoxins, such as colibactin, and encode secretion systems (e.g., T3SS and T6SS) that directly damage host tissues or modulate immune responses. Colibactin induces host DNA damage and is frequently detected in clinical CREC isolates, while hypervirulent clones such as ST410-B5/H24RxC carry high-pathogenicity islands encoding T6SS-associated effectors that enhance intracellular survival and dissemination (Ba et al., 2024; Li et al., 2024b).

CREC can further evade host immunity through the production of outer membrane vesicles and antioxidant systems, contributing to immune modulation and persistence (Söderblom et al., 2005; Kaparakis-Liaskos and Ferrero, 2015; Vanaja et al., 2016; Bielaszewska et al., 2018; Toyofuku et al., 2018; Johnston et al., 2021; Toyofuku et al., 2023). The frequent co-occurrence of resistance and virulence genes may act synergistically to exacerbate disease severity, particularly in immunocompromised hosts. Consequently, CREC infections are often associated with increased clinical complexity, higher mortality rates, and severe complications.

In summary, CREC pathogenicity is multifactorial, involving the coordinated action of virulence determinants, immune evasion strategies, and antimicrobial resistance mechanisms. Elucidating the interplay between resistance and virulence will be critical for understanding CREC transmission and for guiding the development of effective therapeutic and preventive interventions.

## 7 Integrated optimization of CREC prevention and control strategies under the One Health framework

### 7.1 Mechanisms of CREC transmission and critical points for intervention

#### 7.1.1 Humans (hospital and community)

CREC transmission in human populations occurs primarily through nosocomial spread, gastrointestinal colonization of healthy carriers, and horizontal transfer of resistance genes within the gut microbiota. In healthcare settings, CREC can disseminate via patient-healthcare worker contact, contaminated medical devices, and environmental surfaces. In the community, asymptomatic intestinal carriage enables silent transmission through food or direct contact, while horizontal dissemination of *bla*_NDM-5_-IncX3 plasmids among commensal bacteria further amplifies spread.

Critical intervention points include strict implementation of infection prevention and control (IPC) measures in accordance with WHO guidelines, enhanced screening of high-risk patients, and optimized antimicrobial stewardship programs to reduce carbapenem selection pressure (Khabbaz et al., 2014). In parallel, sustained laboratory surveillance using qPCR and whole-genome sequencing is essential for monitoring CREC prevalence and tracking *bla*_NDM-5_ dissemination dynamics (Didelot et al., 2012).

#### 7.1.2 Livestock and poultry farming (animal health)

In animal production systems, CREC transmission is driven by antibiotic misuse, fecal-oral dissemination, and contamination of the food chain. Colonized animals shed resistant bacteria into the environment, facilitating onward transmission to humans through meat products and food-processing chains.

Key interventions include reducing antibiotic use through antibiotic-free or restricted-use farming practices (Holmes et al., 2016), promoting alternatives such as probiotics and antimicrobial peptides (Czaplewski et al., 2016), and implementing policies that reserve specific antimicrobial classes exclusively for human or veterinary use (Årdal et al., 2016). Vaccination strategies and microecological interventions may further reduce intestinal colonization in livestock (Frost et al., 2023; Laxminarayan et al., 2024). Improved manure management and farm hygiene are also critical to limit environmental dissemination.

#### 7.1.3 Environmental contamination (ecological dissemination)

Environmental reservoirs play a central role in CREC dissemination. Hospital and agricultural wastewater serve as major reservoirs of antimicrobial-resistant bacteria and resistance genes, while contaminated soil and water facilitate their persistence and spread. Biofilm formation in environmental matrices further enhances bacterial survival and horizontal gene transfer.

**Figure 3:**
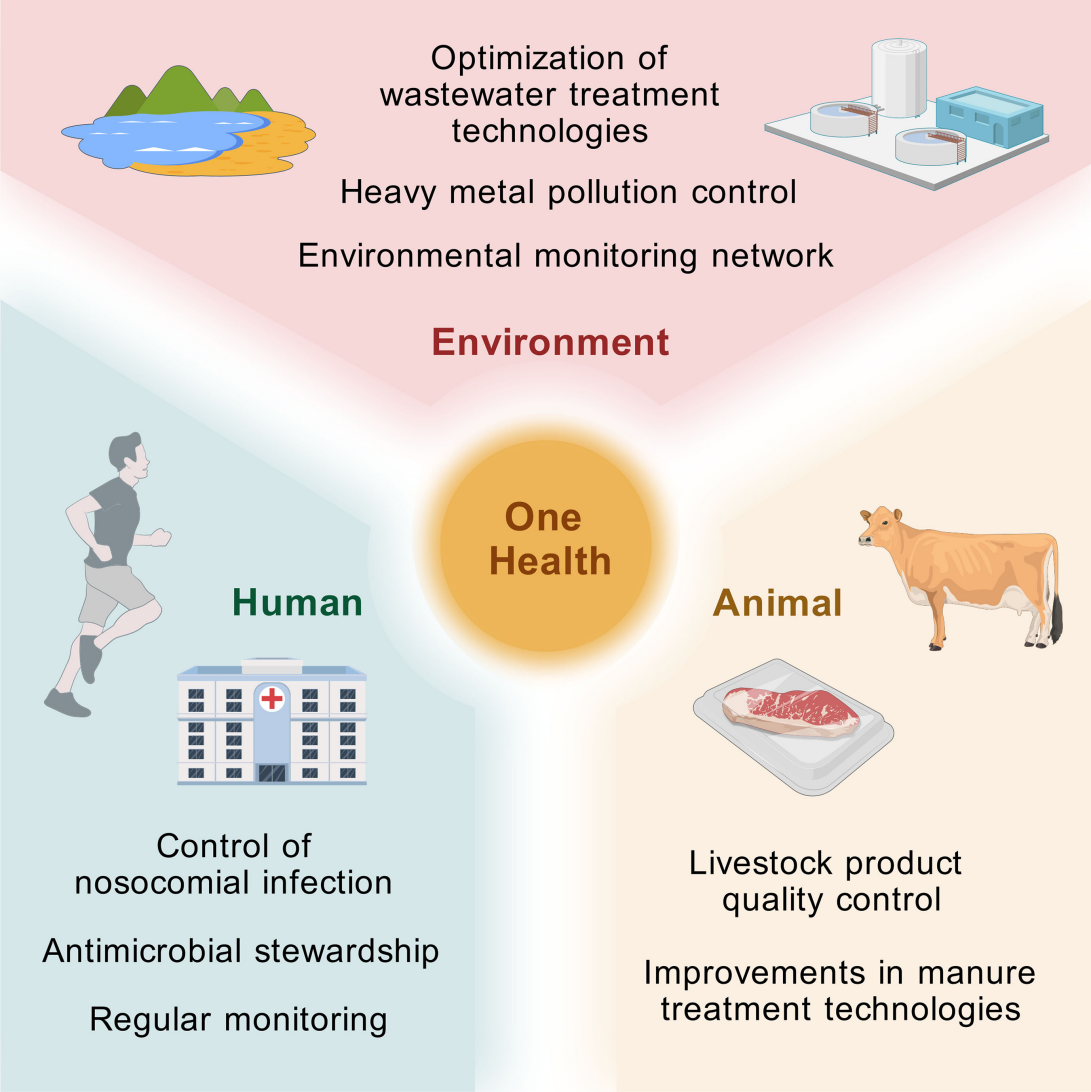
CREC prevention and control strategies under the One Health framework. This image, grounded in the One Health concept, visualizes key points for CREC prevention and control from three perspectives: humans, livestock and poultry, and the environment. For the human population, both hospital and community settings are considered. In hospitals, it is essential to strictly control nosocomial infections and antibiotic use, while in the general population, attention should be given to gastrointestinal colonization by CREC. Regular surveillance of the prevalence and dissemination of antimicrobial resistance genes is also necessary. For livestock and poultry, it is crucial to curb the misuse of antibiotics, ensure the quality of livestock and poultry products from a food chain perspective, improve manure treatment technologies, and reduce the spread of animal-derived resistant bacteria. For the environment, including soil and water sources, hospital and farm wastewater can be hotspots for the accumulation of antimicrobial resistance genes, while wastewater irrigation can lead to soil contamination. The selective pressure exerted by heavy metal ions and their compounds can affect the abundance of resistance genes. Therefore, wastewater treatment technologies should be optimized, and the discharge of heavy metal compounds should be strictly monitored. Based on these considerations, a three-dimensional monitoring network covering humans, livestock and poultry, and the environment should be established to track the transmission and evolution of CREC resistance genes, predict future dissemination trends, and further refine CREC prevention and control strategies. Graphical elements in this figure are adapted from the Generic Diagramming Platform (GDP) (Jiang et al., 2025).

Intervention strategies include optimization of wastewater treatment technologies, such as advanced oxidation, biodegradation, and membrane filtration, to improve removal of resistant bacteria. Reducing environmental discharge of antibiotics and heavy metals, which co-select for resistance, is also essential (Berg et al., 2005; Seiler and Berendonk, 2012; Zhang et al., 2019; Raro et al., 2023). Establishing One Health-oriented environmental surveillance systems enables early detection of resistance trends and risk assessment (**Figure 3**).

### 7.2 Optimization of One Health-based prevention and control strategies

An integrated antimicrobial resistance surveillance system spanning humans, animals, and the environment is fundamental for CREC control. In hospitals, WGS-based monitoring of clinical isolates enables high-resolution tracking of resistance evolution (Didelot et al., 2012). In livestock, systematic screening of fecal samples facilitates early identification of emerging resistance reservoirs. Environmental monitoring of wastewater and soil provides insight into resistance gene abundance and dissemination routes.

Beyond surveillance, strategies to limit the establishment and spread of resistant strains include probiotic-based suppression of intestinal CREC colonization, exploration of phage therapy, and development of targeted biotherapeutics. In animal populations, competitive exclusion approaches, immunological interventions, and improved farm management practices may reduce CREC transmission.

Targeting plasmid-mediated resistance dissemination represents another promising avenue. Interfering with T4SS-mediated conjugation through inhibitors of the VirBR-T4SS regulatory axis, destabilizing resistance plasmids, or introducing incompatible plasmids may reduce transfer frequency of *bla*_NDM-5_-IncX3. In environmental settings, advanced treatments such as graphene oxide, nanosilver, or bioaugmentation strategies may further limit resistance gene spread.

### 7.3 Predictive modeling and risk evaluation

Predictive modeling provides a quantitative framework for assessing CREC transmission risk. Construction of machine learning-based surveillance databases enables identification of key drivers of CREC dissemination and supports early warning systems for outbreaks. SIR-based transmission models can be applied to predict the long-term stability of *_bla_*_NDM-5_-IncX3 plasmids across diverse environments and to assess the impact of antibiotics, heavy metals, and biofilm formation on transmission dynamics. Bayesian network models further facilitate identification of high-risk strains and bacterial hosts with strong dissemination potential.

## 8 Proposed model for cross-species transmission of *bla*_NDM-5_-IncX3 plasmids

**Figure 4:**
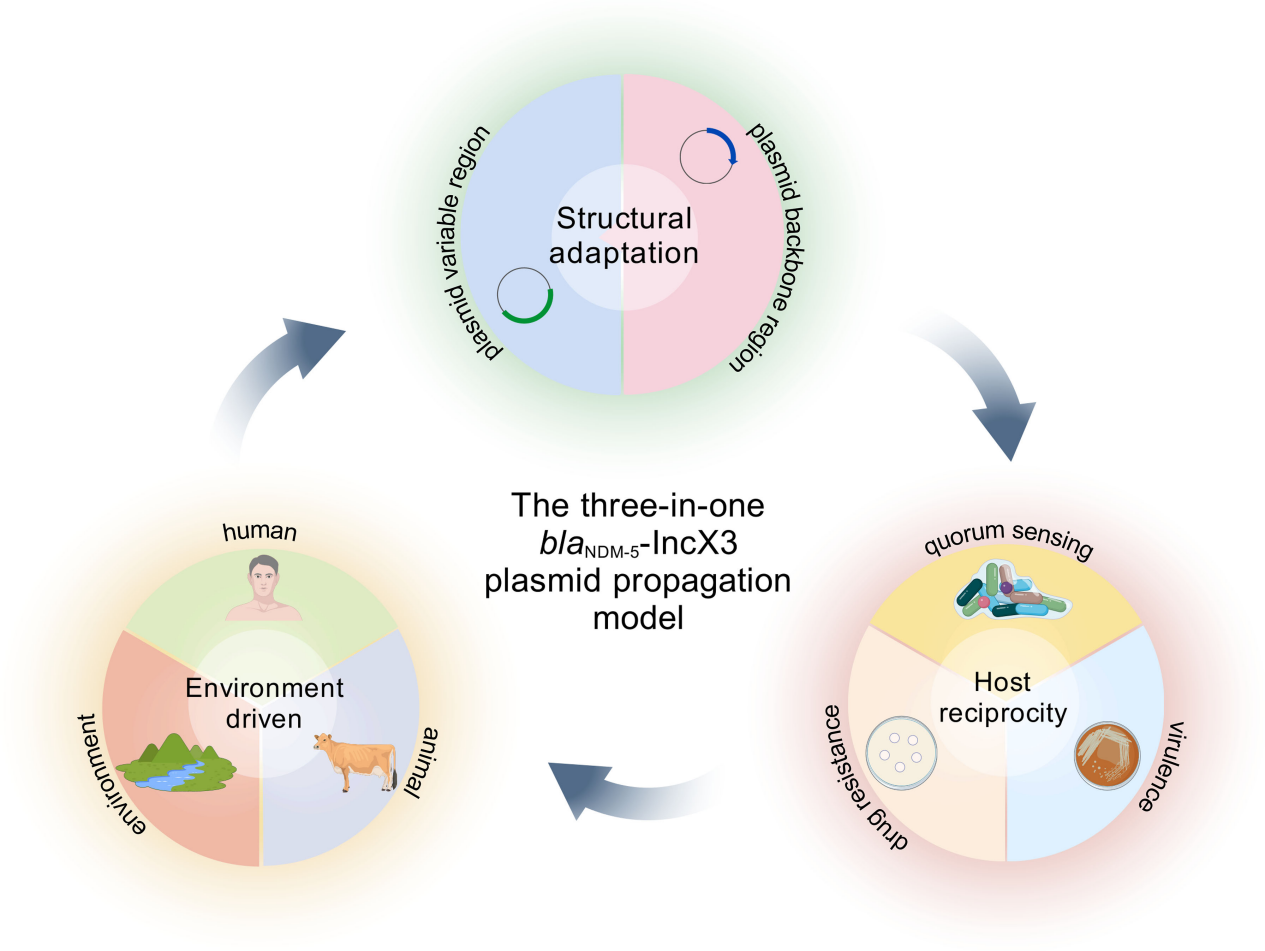
Model for cross-species transmission of *bla*_NDM-5_-IncX3 plasmids. This image presents our innovative “structural adaptation–host reciprocity–environmental driving” triadic model for the cross-species transmission of the *bla*_NDM-5_-IncX3 plasmid. Grounded in the One Health concept, the dissemination and prevalence of CREC resistance plasmids is a multifactorial process that can be summarized into three interconnected components: structural adaptation, host reciprocity, and environmental driving. The variable region of the plasmid serves as the critical site for resistance gene transfer, relying on the complete genetic structure of the backbone to ensure efficient integration and replication—this is considered the first step of the cycle. Subsequently, the host bacterium adapts to the resistance plasmid through metabolic expression, while community-level effects such as biofilm formation further enhance the capacity for synergistic coexistence between the plasmid and its host. These adaptations can manifest as both resistance and virulence phenotypes. Once the host bacterium acquires a certain growth advantage, humans, livestock and poultry, and the environment become key vectors driving its large-scale dissemination. Through the action of these transmission vectors, resistance plasmids diversify their transfer routes, thereby initiating a new cycle of plasmid structural adaptation. Graphical elements in this figure are adapted from the Generic Diagramming Platform (GDP) (Jiang et al., 2025).

Based on current evidence and the One Health framework, we propose a tripartite model to explain the global dissemination of *bla*_NDM-5_-IncX3 plasmids, involving structural adaptation, host reciprocity, and environmental driving forces (**Figure 4**). Structurally, the conserved IncX3 backbone ensures efficient replication and conjugative transfer, while the variable resistance region undergoes dynamic evolution mediated by mobile genetic elements, providing genetic flexibility for resistance dissemination. At the host level, toxin-antitoxin systems, synergy between resistance and virulence traits, and biofilm-associated community effects promote plasmid stability and facilitate plasmid-host co-evolution. Environmentally, antibiotic and heavy metal selection pressures in agriculture, amplification in wastewater systems, and human-environment feedback loops collectively drive cross-species transmission among humans, animals, and environmental reservoirs, forming a self-reinforcing antimicrobial resistance eco-cycle.

The molecular epidemiology of CREC is complex and multifactorial. Effective containment requires coordinated strategies integrating antimicrobial stewardship, rigorous infection control, sustained surveillance, and early intervention across the human-animal-environment interface. Strengthening these measures is essential to curb the ongoing spread of CREC and mitigate its threat to global public health.

## References

[B1] Allen H. K. Donato J. Wang H. H. Cloud-Hansen K. A. Davies J. Handelsman J. (2010). Call of the wild: antibiotic resistance genes in natural environments. Nat. Rev. Microbiol. 8, 251–259. doi: 10.1038/nrmicro2312. PMID: 20190823

[B2] Almakki A. Maure A. Pantel A. Romano-Bertrand S. Masnou A. Marchandin H. . (2017). NDM-5-producing Escherichia coli in an urban river in Montpellier, France. Int. J. Antimicrob. Agents 50, 123–124. doi: 10.1016/j.ijantimicag.2017.04.003. PMID: 28435018

[B3] Antimicrobial Resistance Collaborators (2022). Global burden of bacterial antimicrobial resistance in 2019: a systematic analysis. Lancet 399, 629–655. doi: 10.1016/S0140-6736(21)02724-0, PMID: 35065702 PMC8841637

[B4] Årdal C. Outterson K. Hoffman S. J. Ghafur A. Sharland M. Ranganathan N. . (2016). International cooperation to improve access to and sustain effectiveness of antimicrobials. Lancet 387, 296–307. doi: 10.1016/S0140-6736(15)00470-5, PMID: 26603920

[B5] Arnold B. J. Huang I. T. Hanage W. P. (2021). Horizontal gene transfer and adaptive evolution in bacteria. Nat. Rev. Microbiol. 20, 206–218. doi: 10.1038/s41579-021-00650-4. PMID: 34773098

[B6] Ba X. Guo Y. Moran R. A. Doughty E. L. Liu B. Yao L. . (2024). Global emergence of a hypervirulent carbapenem-resistant Escherichia coli ST410 clone. Nat. Commun. 15, 494. doi: 10.1038/s41467-023-43854-3. PMID: 38216585 PMC10786849

[B7] Baek J. Y. Cho S. Y. Kim S. H. Kang C. I. Peck K. R. Song J. H. . (2019). Plasmid analysis of Escherichia coli isolates from South Korea co-producing NDM-5 and OXA-181 carbapenemases. Plasmid 104, 102417. doi: 10.1016/j.plasmid.2019.102417, PMID: 31150689

[B8] Berg J. Tom-Petersen A. Nybroe O. (2005). Copper amendment of agricultural soil selects for bacterial antibiotic resistance in the field. Lett. Appl. Microbiol. 40, 146–151. doi: 10.1111/j.1472-765x.2004.01650.x. PMID: 15644115

[B9] Bielaszewska M. Marejková M. Bauwens A. Kunsmann-Prokscha L. Mellmann A. Karch H. (2018). Enterohemorrhagic Escherichia coli O157 outer membrane vesicles induce interleukin 8 production in human intestinal epithelial cells by signaling via Toll-like receptors TLR4 and TLR5 and activation of the nuclear factor NF-κB. Int. J. Med. Microbiol. 308, 882–889. doi: 10.1053/j.gastro.2008.12.072. PMID: 29934223

[B10] Bleichenbacher S. Stevens M. J. A. Zurfluh K. Perreten V. Endimiani A. Stephan R. . (2020). Environmental dissemination of carbapenemase-producing Enterobacteriaceae in rivers in Switzerland. Environ. pollut. 265, 115081. doi: 10.1016/j.envpol.2020.115081. PMID: 32806462

[B11] Bliven K. A. Maurelli A. T. (2012). Antivirulence genes: insights into pathogen evolution through gene loss. Infect. Immun. 80, 4061–4070. doi: 10.1128/iai.00740-12. PMID: 23045475 PMC3497401

[B12] Bonomo R. A. Burd E. M. Conly J. Limbago B. M. Poirel L. Segre J. A. . (2018). Carbapenemase-producing organisms: a global scourge. Clin. Infect. Dis. 66, 1290–1297. doi: 10.1093/cid/cix893. PMID: 29165604 PMC5884739

[B13] Brinkac L. M. White R. D'Souza R. Nguyen K. Obaro S. K. Fouts D. E. (2019). Emergence of New Delhi metallo-β-lactamase (NDM-5) in Klebsiella quasipneumoniae from neonates in a Nigerian hospital. mSphere 4, e00685-18. doi: 10.1128/mSphere.00685-18, PMID: 30867330 PMC6416368

[B14] Bush K. Bradford P. A. (2020). Epidemiology of β-lactamase-producing pathogens. Clin. Microbiol. Rev. 33, e00047-19. doi: 10.1128/cmr.00047-19. PMID: 32102899 PMC7048014

[B15] CDC . COVID-19: US impact on antimicrobial resistance, special report 2022. Atlanta, GA: U.S. Department of Health and Human Services, CDC (2022). doi: 10.15620/cdc:117915

[B16] Cole S. D. Peak L. Tyson G. H. Reimschuessel R. Ceric O. Rankin S. C. (2020). New Delhi metallo-β-lactamase-5-producing Escherichia coli in companion animals, United States. Emerg. Infect. Dis. 26, 381–383. doi: 10.3201/eid2602.191221, PMID: 31961309 PMC6986821

[B17] Coque T. M. Cantón R. Pérez-Cobas A. E. Fernández-de-Bobadilla M. D. Baquero F. (2023). Antimicrobial resistance in the global health network: known unknowns and challenges for efficient responses in the 21st century. Microorganisms 11, 1050. doi: 10.3390/microorganisms11041050. PMID: 37110473 PMC10144039

[B18] Costa T. R. D. Harb L. Khara P. Zeng L. Hu B. Christie P. J. (2021). Type IV secretion systems: advances in structure, function, and activation. Mol. Microbiol. 115, 436–452. doi: 10.1111/mmi.14670. PMID: 33326642 PMC8026593

[B19] Croxen M. A. Finlay B. B. (2010). Molecular mechanisms of Escherichia coli pathogenicity. Nat. Rev. Microbiol. 8, 26–38. doi: 10.1038/nrmicro2953. PMID: 19966814

[B20] Czaplewski L. Bax R. Clokie M. Dawson M. Fairhead H. Fischetti V. A. . (2016). Alternatives to antibiotics-a pipeline portfolio review. Lancet Infect. Dis. 16, 239–251. doi: 10.1016/s1473-3099(15)00466-1. PMID: 26795692

[B21] Denamur E. Clermont O. Bonacorsi S. Gordon D. (2021). The population genetics of pathogenic Escherichia coli. Nat. Rev. Microbiol. 19, 37–54. doi: 10.1038/s41579-020-0416-x. PMID: 32826992

[B22] Didelot X. Bowden R. Wilson D. J. Peto T. E. A. Crook D. W. (2012). Transforming clinical microbiology with bacterial genome sequencing. Nat. Rev. Genet. 13, 601–612. doi: 10.1038/nrg3226. PMID: 22868263 PMC5049685

[B23] Di Marcantonio S. Perilli M. Alloggia G. Segatore B. Miconi G. Bruno G. . (2024). Coexistence of bla_NDM-5_, bla_CTX-M-15_, bla_OXA-232_, bla_SHV-182_ genes in multidrug-resistant K. pneumoniae ST437-carrying OmpK36 and OmpK37 porin mutations: First report in Italy. J. Glob. Antimicrob. Resist. 37, 24–27. doi: 10.1016/j.jgar.2024.02.015. PMID: 38408564

[B24] Ding Y. Zhuang H. Zhou J. Xu L. Yang Y. He J. . (2023). Epidemiology and genetic characteristics of carbapenem-resistant Escherichia coli in Chinese intensive care unit analyzed by whole-genome sequencing: a prospective observational study. Microbiol. Spectr. 11, e0401022. doi: 10.1128/spectrum.04010-22. PMID: 36802220 PMC10100791

[B25] Evans D. R. Griffith M. P. Sundermann A. J. Shutt K. A. Saul M. I. Mustapha M. M. . (2020). Systematic detection of horizontal gene transfer across genera among multidrug-resistant bacteria in a single hospital. Elife 9, e53886. doi: 10.7554/elife.53886. PMID: 32285801 PMC7156236

[B26] Frost I. Sati H. Garcia-Vello P. Hasso-Agopsowicz M. Lienhardt C. Gigante V. . (2023). The role of bacterial vaccines in the fight against antimicrobial resistance: an analysis of the preclinical and clinical development pipeline. Lancet Microbe 4, e113–e125. doi: 10.1016/s2666-5247(22)00303-2. PMID: 36528040 PMC9892012

[B27] Fulsundar S. Harms K. Flaten G. E. Johnsen P. J. Chopade B. A. Nielsen K. M. (2014). Gene transfer potential of outer membrane vesicles of Acinetobacter baylyi and effects of stress on vesiculation. Appl. Environ. Microbiol. 80, 3469–3483. doi: 10.1128/aem.04248-13. PMID: 24657872 PMC4018862

[B28] GBD 2021 Antimicrobial Resistance Collaborators (2024). Global burden of bacterial antimicrobial resistance 1990-2021: a systematic analysis with forecasts to 2050. Lancet 404, 1199–1226. doi: 10.1016/S0140-6736(24)01867-1, PMID: 39299261 PMC11718157

[B29] González-Espinosa F. Di Pilato V. Calabrese L. Costa E. Costa A. Gutkind G. . (2024). Integral genomic description of bla_NDM-5_-harbouring plasmids recovered from Enterobacterales in Argentina. J. Glob. Antimicrob. Resist. 39, 224–226. doi: 10.1016/j.jgar.2024.10.258. PMID: 39477069

[B30] Guo X. Chen R. Wang Q. Li C. Ge H. Qiao J. . (2022). Global prevalence, characteristics, and future prospects of IncX3 plasmids: a review. Front. Microbiol. 13, 979558. doi: 10.3389/fmicb.2022.979558. PMID: 36147856 PMC9485871

[B31] Harris P. N. Tambyah P. A. Paterson D. L. (2015). β-lactam and β-lactamase inhibitor combinations in the treatment of extended-spectrum β-lactamase producing Enterobacteriaceae: time for a reappraisal in the era of few antibiotic options? Lancet Infect. Dis. 15, 475–485. doi: 10.1016/S1473-3099(14)70950-8, PMID: 25716293

[B32] Hickman R. A. Leangapichart T. Lunha K. Jiwakanon J. Angkititrakul S. Magnusson U. . (2021). Exploring the antibiotic resistance burden in livestock, livestock handlers and their non-livestock handling contacts: a one health perspective. Front. Microbiol. 12, 651461. doi: 10.3389/fmicb.2021.651461. PMID: 33959112 PMC8093850

[B33] Ho P. L. Wang Y. Liu M. C. Lai E. L. Law P. Y. Cao H. . (2018). IncX3 epidemic plasmid carrying bla_NDM-5_ in Escherichia coli from swine in multiple geographic areas in China. Antimicrob. Agents Chemother. 62, e02295-17. doi: 10.3389/fmicb.2018.02272. PMID: 29311058 PMC5826167

[B34] Holmes A. H. Moore L. S. Sundsfjord A. Steinbakk M. Regmi S. Karkey A. . (2016). Understanding the mechanisms and drivers of antimicrobial resistance. Lancet 387, 176–187. doi: 10.1016/s0140-6736(15)00473-0. PMID: 26603922

[B35] Hong J. S. Song W. Jeong S. H. (2020). Molecular characteristics of NDM-5-producing Escherichia coli from a cat and a dog in South Korea. Microb. Drug Resist. 26, 1005–1008. doi: 10.1089/mdr.2019.0382. PMID: 32043911

[B36] Hooban B. Joyce A. Fitzhenry K. Chique C. Morris D. (2020). The role of the natural aquatic environment in the dissemination of extended spectrum beta-lactamase and carbapenemase encoding genes: A scoping review. Water Res. 180, 115880. doi: 10.1016/j.watres.2020.115880. PMID: 32438141

[B37] Hornsey M. Phee L. Wareham D. W. (2011). A novel variant, NDM-5, of the New Delhi metallo-β-lactamase in a multidrug-resistant Escherichia coli ST648 isolate recovered from a patient in the United Kingdom. Antimicrob. Agents Chemother. 55, 5952–5954. doi: 10.1128/AAC.05108-11, PMID: 21930874 PMC3232805

[B38] Huang J. Lv C. Li M. Rahman T. Chang Y. F. Guo X. . (2024). Carbapenem-resistant Escherichia coli exhibit diverse spatiotemporal epidemiological characteristics across the globe. Commun. Biol. 7, 51. doi: 10.1016/j.jmii.2015.02.064. PMID: 38184739 PMC10771496

[B39] Ilangovan A. Connery S. Waksman G. (2015). Structural biology of the Gram-negative bacterial conjugation systems. Trends Microbiol. 23, 301–310. doi: 10.1016/j.tim.2015.02.012. PMID: 25825348

[B40] Jamin C. De Koster S. van Koeveringe S. De Coninck D. Mensaert K. De Bruyne K. . (2021). Harmonization of whole-genome sequencing for outbreak surveillance of Enterobacteriaceae and Enterococci. Microb. Genom. 7, 567. doi: 10.1099/mgen.0.000567. PMID: 34279213 PMC8477410

[B41] Jiang S. Li H. Zhang L. Mu W. Zhang Y. Chen T. . (2025). Generic Diagramming Platform (GDP): a comprehensive database of high-quality biomedical graphics. Nucleic Acids Res. 53, D1670–D1676. doi: 10.1093/nar/gkae973. PMID: 39470721 PMC11701665

[B42] Jiang Y. Wang Y. Hua X. Qu Y. Peleg A. Y. Yu Y. (2020). Pooled plasmid sequencing reveals the relationship between mobile genetic elements and antimicrobial resistance genes in clinically isolated Klebsiella pneumoniae. Genomics Proteomics Bioinf. 18, 539–548. doi: 10.1016/j.gpb.2020.12.002. PMID: 33385612 PMC8377239

[B43] Johnson T. J. Bielak E. M. Fortini D. Hansen L. H. Hasman H. Debroy C. . (2012). Expansion of the IncX plasmid family for improved identification and typing of novel plasmids in drug-resistant Enterobacteriaceae. Plasmid 68, 43–50. doi: 10.1016/j.plasmid.2012.03.001. PMID: 22470007

[B44] Johnston E. L. Heras B. Kufer T. A. Kaparakis-Liaskos M. (2021). Detection of bacterial membrane vesicles by NOD-like receptors. Int. J. Mol. Sci. 22, 1005. doi: 10.3390/ijms22031005. PMID: 33498269 PMC7863931

[B45] Johnston C. Martin B. Fichant G. Polard P. Claverys J. P. (2014). Bacterial transformation: distribution, shared mechanisms and divergent control. Nat. Rev. Microbiol. 12, 181–196. doi: 10.1038/nrmicro3199. PMID: 24509783

[B46] Kaparakis-Liaskos M. Ferrero R. L. (2015). Immune modulation by bacterial outer membrane vesicles. Nat. Rev. Immunol. 15, 375–387. doi: 10.1038/nri3837. PMID: 25976515

[B47] Kaper J. B. Nataro J. P. Mobley H. L. (2004). Pathogenic Escherichia coli. Nat. Rev. Microbiol. 2, 123–140. doi: 10.1016/j.ijmm.2005.06.008. PMID: 15040260

[B48] Kayama S. Yu L. Kawakami S. . (2022). Emergence of bla_NDM-5_-carrying Klebsiella aerogenes in Japan. Microbiol. Spectr. 10, e0222221. doi: 10.1128/aac.01062-13. PMID: 35658578 PMC9241950

[B49] Khabbaz R. F. Moseley R. R. Steiner R. J. Levitt A. M. Bell B. P. (2014). Challenges of infectious diseases in the USA. Lancet 384, 53–63. doi: 10.1016/s0140-6736(14)60890-4. PMID: 24996590 PMC7137922

[B50] Kizny Gordon A. Phan H. T. T. Lipworth S. I. Cheong E. Gottlieb T. George S. . (2020). Genomic dynamics of species and mobile genetic elements in a prolonged blaIMP-4-associated carbapenemase outbreak in an Australian hospital. J. Antimicrob. Chemother. 75, 873–882. doi: 10.1093/jac/dkz526. PMID: 31960024 PMC7069471

[B51] Krishnaraju M. Kamatchi C. Jha A. K. Devasena N. Vennila R. Sumathi G. . (2015). Complete sequencing of an IncX3 plasmid carrying bla_NDM-5_ allele reveals an early stage in the dissemination of the bla_NDM_ gene. Indian J. Med. Microbiol. 33, 30–38. doi: 10.4103/0255-0857.148373. PMID: 25559999

[B52] Ladner J. T. Grubaugh N. D. Pybus O. G. Andersen K. G. (2019). Precision epidemiology for infectious disease control. Nat. Med. 25, 206–211. doi: 10.1038/s41591-019-0345-2. PMID: 30728537 PMC7095960

[B53] Laxminarayan R. Impalli I. Rangarajan R. Cohn J. Ramjeet K. Trainor B. W. . (2024). Expanding antibiotic, vaccine, and diagnostics development and access to tackle antimicrobial resistance. Lancet 403, 2534–2550. doi: 10.1016/s0140-6736(24)00878-x. PMID: 38797178

[B54] Laxminarayan R. Matsoso P. Pant S. Brower C. Røttingen J. A. Klugman K. . (2016). Access to effective antimicrobials: a worldwide challenge. Lancet 387, 168–175. doi: 10.1016/s0140-6736(15)00474-2. PMID: 26603918

[B55] Li X. Fu Y. Shen M. Huang D. Du X. Hu Q. . (2018). Dissemination of bla_NDM-5_ gene via an IncX3-type plasmid among non-clonal Escherichia coli in China. Antimicrob. Resist. Infect. Control 7, 59. doi: 10.14711/thesis-991012565062103412 29713466 PMC5918551

[B56] Li L. Gao Y. Wang L. Lu F. Ji Q. Zhang Y. . (2024). The effects of NDM-5 on Escherichia coli and the screening of interacting proteins. Front. Microbiol. 15, 1328572. doi: 10.3389/fmicb.2024.1328572. PMID: 38348193 PMC10861311

[B57] Li Y. Sun X. Dong N. Wang Z. Li R. (2024a). Global distribution and genomic characteristics of carbapenemase-producing Escherichia coli among humans, 2005-2023. Drug Resist. Update 72, 101031. doi: 10.1016/j.drup.2023.101031. PMID: 38071860

[B58] Li Y. Sun X. Xiao X. Wang Z. Li R. (2023). Global distribution and genomic characteristics of tet(X)-positive Escherichia coli among humans, animals, and the environment. Sci. Total Environ. 887, 164148. doi: 10.1016/j.scitotenv.2023.164148. PMID: 37187393

[B59] Li Y. Tang M. Dai X. Zhou Y. Zhang Z. Qiu Y. . (2021). Whole-genomic analysis of NDM-5-producing Enterobacteriaceae recovered from an urban river in China. Infect. Drug Resist. 14, 4427–4440. doi: 10.2147/idr.s330787. PMID: 34737583 PMC8559237

[B60] Li F. Ye K. Li X. Ye L. Guo L. Wang L. . (2021). Genetic characterization of carbapenem-resistant Escherichia coli from China, 2015-2017. BMC Microbiol. 21, 248. doi: 10.1186/s12866-021-02307-x. PMID: 34535075 PMC8449468

[B61] Li Y. Zhang Y. Sun X. Wu Y. Yan Z. Ju X. . (2024b). National genomic epidemiology investigation revealed the spread of carbapenem-resistant Escherichia coli in healthy populations and the impact on public health. Genome Med. 16, 57. doi: 10.1186/s13073-024-01310-x. PMID: 38627827 PMC11020349

[B62] Lian Z. J. Phan M. D. Hancock S. J. Nhu N. T. K. Paterson D. L. Schembri M. A. (2023). Genetic basis of I-complex plasmid stability and conjugation. PloS Genet. 19, e1010773. doi: 10.1371/journal.pgen.1010773. PMID: 37347771 PMC10286972

[B63] Liu B. Guo Y. Liu N. Wang J. Li F. Yao L. . (2021). In silico evolution and comparative genomic analysis of IncX3 plasmids isolated from China over ten years. Front. Microbiol. 12, 725391. doi: 10.3389/fmicb.2021.725391. PMID: 34925253 PMC8681339

[B64] Liu Y. Y. Li T. Yue H. Yue C. Lu L. Chen J. . (2023). Occurrence and characterization of NDM-5-producing Escherichia coli from retail eggs. Front. Microbiol. 14, 1281838. doi: 10.3389/fmicb.2023.1281838. PMID: 38075903 PMC10701905

[B65] Liu B. Shui L. Zhou K. Jiang Y. Li X. Guan J. . (2020). Impact of plasmid-encoded H-NS-like protein on bla_NDM-1_-bearing IncX3 plasmid in Escherichia coli. J. Infect. Dis. 221, S229–S236. doi: 10.1093/infdis/jiz567. PMID: 32176784

[B66] Liu Z. Xiao X. Li Y. Liu Y. Li R. Wang Z. (2019). Emergence of IncX3 plasmid-harboring bla_NDM-5_ dominated by Escherichia coli ST48 in a goose farm in Jiangsu, China. Front. Microbiol. 10. doi: 10.3389/fmicb.2019.02002. PMID: 31551956 PMC6737504

[B67] Logan L. K. Weinstein R. A. (2017). The epidemiology of carbapenem-resistant Enterobacteriaceae: The impact and evolution of a global menace. J. Infect. Dis. 215, S28–S36. doi: 10.1093/infdis/jiw282. PMID: 28375512 PMC5853342

[B68] Long H. Feng Y. Ma K. Liu L. McNally A. Zong Z. (2019). The co-transfer of plasmid-borne colistin-resistant genes mcr-1 and mcr-3.5, the carbapenemase gene bla_NDM-5_ and the 16S methylase gene rmtB from Escherichia coli. Sci. Rep. 9, 696. doi: 10.1038/s41598-018-37125-1. PMID: 30679636 PMC6346057

[B69] Lu Y. L. Dai Z. Y. Chen L. Y. Chen Y. F. Sun D. C. Yang H. . (2020). Antimicrobial resistance of two avian-origin carbapenem-resistant Escherichia coli ST10 and ST354 strains mediated by the bla_NDM-5_ gene. Microbiol. China 47, 1837–1846. doi: 10.13344/j.microbiol.china.190856

[B70] Ma Z. Wang B. Zeng D. Ding H. Zeng Z. (2024). Rapid dissemination of bla_NDM-5_ gene among carbapenem-resistant Escherichia coli isolates in a yellow-feather broiler farm via multiple plasmid replicon. Pathogens 13, 387. doi: 10.1016/j.cjca.2020.03.042. PMID: 38787239 PMC11124502

[B71] Ma T. Xie N. Gao Y. Fu J. Tan C. E. Yang Q. E. . (2024). VirBR, a transcription regulator, promotes IncX3 plasmid transmission, and persistence of bla_NDM-5_ in zoonotic bacteria. Nat. Commun. 15, 5498. doi: 10.1038/s41467-024-49800-1. PMID: 38944647 PMC11214620

[B72] Macé K. Vadakkepat A. K. Redzej A. Lukoyanova N. Oomen C. Braun N. . (2022). Cryo-EM structure of a type IV secretion system. Nature 607, 191–196. doi: 10.1038/s41586-022-04859-y, PMID: 35732732 PMC9259494

[B73] Macesic N. Dennis A. Hawkey J. Vezina B. Wisniewski J. A. Cottingham H. . (2024). Genomic investigation of multispecies and multivariant bla_NDM_ outbreak reveals key role of horizontal plasmid transmission. Infect. Control Hosp. Epidemiol. 45, 709–716. doi: 10.1017/ice.2024.8. PMID: 38344902 PMC11102827

[B74] Macesic N. Uhlemann A. C. Peleg A. Y. (2025). Multidrug-resistant Gram-negative bacterial infections. Lancet 405, 257–272. doi: 10.1016/s0140-6736(24)02081-6. PMID: 39826970

[B75] Marchetti V. M. Bitar I. Mercato A. Nucleo E. Bonomini A. Pedroni P. . (2020). Complete nucleotide sequence of plasmids of two Escherichia coli strains carrying bla_NDM-5_ and bla_NDM-5_ and bla_OXA-181_ from the same patient. Front. Microbiol. 10, 3095. doi: 10.3389/fmicb.2019.03095. PMID: 32038543 PMC6985152

[B76] Mazel D. (2006). Integrons: agents of bacterial evolution. Nat. Rev. Microbiol. 4, 608–620. doi: 10.1038/nrmicro1462. PMID: 16845431

[B77] Miller W. R. Arias C. A. (2024). ESKAPE pathogens: antimicrobial resistance, epidemiology, clinical impact and therapeutics. Nat. Rev. Microbiol. 22, 598–616. doi: 10.1038/s41579-024-01054-w. PMID: 38831030 PMC13147291

[B78] Moon B. Y. Ali M. S. Kim S. Kang H. S. Kang Y. J. Kim J. M. . (2024). Prevalence and molecular characteristics of carbapenem-resistant Escherichia coli isolated from dogs in South Korea. J. Vet. Sci. 25, e67. doi: 10.4142/jvs.24164. PMID: 39363655 PMC11450394

[B79] Moon B. Y. Ali M. S. Kwon D. H. Heo Y. E. Hwang Y. J. Kim J. I. . (2023). Antimicrobial resistance in Escherichia coli isolated from healthy dogs and cats in South Korea, 2020-2022. Antibiotics (Basel) 13, 27. doi: 10.1111/j.1863-2378.2011.01450.x. PMID: 38247586 PMC10812631

[B80] Naas T. Oueslati S. Bonnin R. A. Dabos M. L. Zavala A. Dortet L. . (2017). Beta-lactamase database (BLDB) - structure and function. J. Enzyme Inhib. Med. Chem. 32, 917–919. doi: 10.1080/14756366.2017.1344235. PMID: 28719998 PMC6445328

[B81] Ni S. Li B. Tang K. Yao J. Wood T. K. Wang P. . (2021). Conjugative plasmid-encoded toxin-antitoxin system PrpT/PrpA directly controls plasmid copy number. Proc. Natl. Acad. Sci. U.S.A. 118, e2011577118. doi: 10.1073/pnas.2011577118. PMID: 33483419 PMC7848731

[B82] Nordmann P. Poirel L. (2019). Epidemiology and diagnostics of carbapenem resistance in Gram-negative bacteria. Clin. Infect. Dis. 69, S521–S528. doi: 10.1093/cid/ciz824. PMID: 31724045 PMC6853758

[B83] Okeke I. N. de Kraker M. E. A. Van Boeckel T. P. Kumar C. K. Schmitt H. Gales A. C. . (2024). The scope of the antimicrobial resistance challenge. Lancet 403, 2426–2438. doi: 10.1016/s0140-6736(24)00876-6. PMID: 38797176

[B84] Pál T. Ghazawi A. Darwish D. Villa L. Carattoli A. Hashmey R. . (2017). Characterization of NDM-7 carbapenemase-producing Escherichia coli isolates in the Arabian Peninsula. Microb. Drug Resist. 23, 871–878. doi: 10.1089/mdr.2016.0216, PMID: 28156193

[B85] Peirano G. Chen L. Nobrega D. Finn T. J. Kreiswirth B. N. DeVinney R. . (2022). Genomic epidemiology of global carbapenemase-producing Escherichia coli, 2015-2017. Emerg. Infect. Dis. 28, 924–931. doi: 10.3201/eid2805.212535. PMID: 35451367 PMC9045447

[B86] Raro O. H. F. Poirel L. Nordmann P. (2023). Effect of zinc oxide and copper sulfate on antibiotic resistance plasmid transfer in Escherichia coli. Microorganisms 11, 2880. doi: 10.3390/microorganisms11122880. PMID: 38138025 PMC10745819

[B87] Ravi A. Valdés-Varela L. Gueimonde M. Rudi K. (2018). Transmission and persistence of IncF conjugative plasmids in the gut microbiota of full-term infants. FEMS Microbiol. Ecol. 94, 1–9. doi: 10.1093/femsec/fix158. PMID: 29161377

[B88] Sabour S. Huang J. Y. Bhatnagar A. Gilbert S. E. Karlsson M. Lonsway D. . (2021). Detection and characterization of targeted carbapenem-resistant health care-associated threats: findings from the Antibiotic Resistance Laboratory Network, 2017 to 2019. Antimicrob. Agents Chemother. 65, e0110521. doi: 10.1128/aac.01105-21. PMID: 34570648 PMC8597727

[B89] Seiler C. Berendonk T. U. (2012). Heavy metal driven co-selection of antibiotic resistance in soil and water bodies impacted by agriculture and aquaculture. Front. Microbiol. 3. doi: 10.3389/fmicb.2012.00399. PMID: 23248620 PMC3522115

[B90] Sekizuka T. Inamine Y. Segawa T. Kuroda M. (2019). Characterization of NDM-5- and CTX-M-55-coproducing Escherichia coli GSH8M-2 isolated from the effluent of a wastewater treatment plant in Tokyo Bay. Infect. Drug Resist. 12, 2243–2249. doi: 10.2147/IDR.S215273, PMID: 31413601 PMC6662510

[B91] Shen Z. Hu Y. Sun Q. Hu F. Zhou H. Shu L. . (2018). Emerging carriage of NDM-5 and MCR-1 in Escherichia coli from healthy people in multiple regions in China: a cross sectional observational study. EClinicalMedicine 6, 11–20. doi: 10.1016/j.eclinm.2018.11.003. PMID: 31193653 PMC6537561

[B92] Söderblom T. Oxhamre C. Wai S. N. Uhlén P. Aperia A. Uhlin B. E. . (2005). Effects of the Escherichia coli toxin cytolysin A on mucosal immunostimulation via epithelial Ca^2+^ signalling and Toll-like receptor 4. Cell. Microbiol. 7, 779–788. doi: 10.3233/978-1-60750-031-5-1923. PMID: 15888081

[B93] Sokurenko E. V. Chesnokova V. Dykhuizen D. E. Ofek I. Wu X. R. Krogfelt K. A. . (1998). Pathogenic adaptation of Escherichia coli by natural variation of the FimH adhesin. Proc. Natl. Acad. Sci. U.S.A. 95, 8922–8926. doi: 10.1073/pnas.95.15.8922. PMID: 9671780 PMC21178

[B94] Sokurenko E. V. Hasty D. L. Dykhuizen D. E. (1999). Pathoadaptive mutations: gene loss and variation in bacterial pathogens. Trends Microbiol. 7, 191–195. doi: 10.1016/s0966-842x(99)01493-6. PMID: 10354593

[B95] Suleyman G. Shallal A. Ruby A. Chami E. Gubler J. McNamara S. . (2024). Use of whole genomic sequencing to detect New Delhi metallo-B-lactamase (NDM)-producing Escherichia coli outbreak associated with endoscopic procedures. Infect. Control Hosp. Epidemiol. 45, 965–972. doi: 10.1017/ice.2024.36. PMID: 38495009

[B96] Sun P. Xia W. Liu G. Huang X. Tang C. Liu C. . (2019). Characterization of bla_NDM-5_-positive Escherichia coli prevalent in a university hospital in eastern China. Infect. Drug Resist. 12, 3029–3038. doi: 10.1007/bf00241911. PMID: 31576153 PMC6767761

[B97] Sun J. Yang R. S. Zhang Q. Feng Y. Fang L. X. Xia J. . (2016). Co-transfer of bla_NDM-5_ and mcr-1 by an IncX3-X4 hybrid plasmid in Escherichia coli. Nat. Microbiol. 1, 16176. doi: 10.1038/nmicrobiol.2016.176, PMID: 27668643

[B98] Tamma P. D. Heil E. L. Justo J. A. Mathers A. J. Satlin M. J. Bonomo R. A. (2024). Infectious Diseases Society of America 2024 guidance on the treatment of antimicrobial-resistant Gram-negative infections. Clin. Infect. Dis., ciae403. doi: 10.1093/cid/ciae403. PMID: 39108079

[B99] Tashiro Y. Hasegawa Y. Shintani M. Takaki K. Ohkuma M. Kimbara K. . (2017). Interaction of bacterial membrane vesicles with specific species and their potential for delivery to target cells. Front. Microbiol. 8, 571. doi: 10.3389/fmicb.2017.00571. PMID: 28439261 PMC5383704

[B100] Tenaillon O. Skurnik D. Picard B. Denamur E. (2010). The population genetics of commensal Escherichia coli. Nat. Rev. Microbiol. 8, 207–217. doi: 10.1038/nrmicro2298. PMID: 20157339

[B101] Teng L. Feng M. Liao S. Zheng Z. Jia C. Zhou X. . (2023). A cross-sectional study of companion animal-derived multidrug-resistant Escherichia coli in Hangzhou, China. Microbiol. Spectr. 11, e0211322. doi: 10.1128/spectrum.02113-22. PMID: 36840575 PMC10100847

[B102] Theuretzbacher U. (2025). The global resistance problem and the clinical antibacterial pipeline. Nat. Rev. Microbiol. 23, 491–508. doi: 10.1038/s41579-025-01169-8. PMID: 40210708

[B103] Theuretzbacher U. Jumde R. P. Hennessy A. Cohn J. Piddock L. J. V. (2025). Global health perspectives on antibacterial drug discovery and the preclinical pipeline. Nat. Rev. Microbiol. 23, 474–490. doi: 10.1038/s41579-025-01167-w. PMID: 40148602

[B104] Tian D. Wang B. Zhang H. Pan F. Wang C. Shi Y. . (2020). Dissemination of the bla_NDM-5_ gene via IncX3-type plasmid among Enterobacteriaceae in children. mSphere 5, e00699-19. doi: 10.21013/jems.v8.n1.p2 PMC6952193 31915216

[B105] Toyofuku M. Nomura N. Eberl L. (2018). Types and origins of bacterial membrane vesicles. Nat. Rev. Microbiol. 17, 13–24. doi: 10.1038/s41579-018-0112-2. PMID: 30397270

[B106] Toyofuku M. Schild S. Kaparakis-Liaskos M. Eberl L. (2023). Composition and functions of bacterial membrane vesicles. Nat. Rev. Microbiol. 21, 415–430. doi: 10.1038/s41579-023-00875-5. PMID: 36932221

[B107] Tran F. Boedicker J. Q. (2017). Genetic cargo and bacterial species set the rate of vesicle-mediated horizontal gene transfer. Sci. Rep. 7, 8813. doi: 10.1038/s41598-017-07447-7. PMID: 28821711 PMC5562762

[B108] Tsilipounidaki K. Athanasakopoulou Z. Billinis C. Miriagou V. Petinaki E. (2022). Letter to the editor: Importation of the first bovine ST361 New Delhi metallo-5 positive Escherichia coli in Greece. Microb. Drug Resist. 28, 386–387. doi: 10.1089/mdr.2021.0243. PMID: 34935518 PMC8968834

[B109] Turton J. F. Pike R. Perry C. Jenkins C. Turton J. A. Meunier D. . (2022). Wide distribution of Escherichia coli carrying IncF plasmids containing bla_NDM-5_ and rmtB resistance genes from hospitalized patients in England. J. Med. Microbiol. 71, 1–9. doi: 10.1099/jmm.0.001569. PMID: 35925786

[B110] Vanaja S. K. Russo A. J. Behl B. Banerjee I. Yankova M. Deshmukh S. D. . (2016). Bacterial outer membrane vesicles mediate cytosolic localization of LPS and caspase-11 activation. Cell 165, 1106–1119. doi: 10.1016/j.cell.2016.04.015. PMID: 27156449 PMC4874922

[B111] van Duin D. Arias C. A. Komarow L. Chen L. Hanson B. M. Weston G. . (2020). Molecular and clinical epidemiology of carbapenem-resistant Enterobacterales in the USA (CRACKLE-2): a prospective cohort study. Lancet Infect. Dis. 20, 731–741. doi: 10.1016/s1473-3099(19)30755-8. PMID: 32151332 PMC7473597

[B112] Wailan A. M. Paterson D. L. Caffery M. Sowden D. Sidjabat H. E. (2015). Draft genome sequence of NDM-5-producing Escherichia coli sequence type 648 and genetic context of bla_NDM-5_ in Australia. Genome Announc 3, e00194-15. doi: 10.1128/genomea.00194-15. PMID: 25858833 PMC4392145

[B113] Wang D. Berglund B. Li Q. Shangguan X. Li J. Liu F. . (2023). Transmission of clones of carbapenem-resistant Escherichia coli between a hospital and an urban wastewater treatment plant. Environ. pollut. 336, 122455. doi: 10.1016/j.envpol.2023.122455. PMID: 37633440

[B114] Wang Y. Zhang R. Li J. Wu Z. Yin W. Schwarz S. . (2017). Comprehensive resistome analysis reveals the prevalence of NDM and MCR-1 in Chinese poultry production. Nat. Microbiol. 2, 16260. doi: 10.1038/nmicrobiol.2016.260. PMID: 28165472

[B115] Wang M. Zhang Z. Sun Z. Wang X. Zhu J. Jiang M. . (2025). The emergence of highly resistant and hypervirulent Escherichia coli ST405 clone in a tertiary hospital over 8 years. Emerging Microbes Infections 14, 2479048. doi: 10.1080/22221751.2025.2479048. PMID: 40071947 PMC11934165

[B116] Wang M. G. Zhang R. M. Wang L. L. Sun R. Y. Bai S. C. Han L. . (2021). Molecular epidemiology of carbapenemase-producing Escherichia coli from duck farms in south-east coastal China. J. Antimicrob. Chemother. 76. doi: 10.3389/fcimb.2021.661218. PMID: 33057710

[B117] Wang Q. Zhou L. Chen X. Yao J. Sun X. Peng K. . (2025). Global emergence and transmission dynamics of carbapenemase-producing Citrobacter freundii sequence type 22 high-risk international clone: a retrospective, genomic, epidemiological study. Lancet Microbe 6, 101149. doi: 10.1016/j.lanmic.2025.101149. PMID: 40683282

[B118] WHO (2024). WHO bacterial priority pathogens list, 2024: Bacterial pathogens of public health importance to guide research, development and strategies to prevent and control antimicrobial resistance. 1–72.

[B119] Wilson B. M. El Chakhtoura N. G. Patel S. Saade E. Donskey C. J. Bonomo R. A. . (2017). Carbapenem-resistant Enterobacter cloacae in patients from the US Veterans Health Administration, 2006-2015. Emerg. Infect. Dis. 23, 878–880. doi: 10.3201/eid2305.162034. PMID: 28418318 PMC5403041

[B120] Wright G. D. (2007). The antibiotic resistome: the nexus of chemical and genetic diversity. Nat. Rev. Microbiol. 5, 175–186. doi: 10.1038/nrmicro1614. PMID: 17277795

[B121] Wu W. Feng Y. Tang G. Qiao F. McNally A. Zong Z. (2019). NDM metallo-β-lactamases and their bacterial producers in health care settings. Clin. Microbiol. Rev. 32, e00115-18. doi: 10.1128/cmr.00115-18. PMID: 30700432 PMC6431124

[B122] Xiang G. Zhao Z. Zhang S. Cai Y. He Y. Zeng J. . (2024). Porin deficiency or plasmid copy number increase mediated carbapenem-resistant Escherichia coli resistance evolution. Emerg. Microbes Infect. 13, 2352432. doi: 10.1080/22221751.2024.2352432. PMID: 38712634 PMC11107853

[B123] Xu J. Guo H. Li L. He F. (2023). Molecular epidemiology and genomic insights into the transmission of carbapenem-resistant NDM-producing Escherichia coli. Comput. Struct. Biotechnol. J. 21, 847–855. doi: 10.1016/j.csbj.2023.01.004. PMID: 36698971 PMC9842800

[B124] Yang J. Lu Y. Yu J. Cai X. Wang C. Lv L. . (2025). Comprehensive analysis of Enterobacteriaceae IncX plasmids reveals robust conjugation regulators PrfaH, H-NS, and conjugation-fitness tradeoff. Commun. Biol. 8, 363. doi: 10.5220/0014348500004918 40038536 PMC11880322

[B125] Yang Q. E. Ma X. Zeng L. Wang Q. Li M. Teng L. . (2024). Interphylum dissemination of NDM-5-positive plasmids in hospital wastewater from Fuzhou, China: a single-centre, culture-independent, plasmid transmission study. Lancet Microbe 5, e13–e23. doi: 10.1016/s2666-5247(23)00227-6. PMID: 38006896

[B126] Yong D. Toleman M. A. Giske C. G. Cho H. S. Sundman K. Lee K. . (2009). Characterization of a new metallo-beta-lactamase gene, bla(NDM-1), and a novel erythromycin esterase gene carried on a unique genetic structure in Klebsiella pneumoniae sequence type 14 from India. Antimicrob. Agents Chemother. 53, 5046–5054. doi: 10.1128/aac.00774-09. PMID: 19770275 PMC2786356

[B127] Yuan P. B. Dai L. T. Zhang Q. K. Zhong Y. X. Liu W. T. Yang L. . (2024). Global emergence of double and multi-carbapenemase producing organisms: epidemiology, clinical significance, and evolutionary benefits on antimicrobial resistance and virulence. Microbiol. Spectr. 12, e0000824. doi: 10.1128/spectrum.00008-24. PMID: 38860788 PMC11218513

[B128] Zeng S. Huang Y. Zhang X. Fu L. Sun Z. Li X. (2023). Molecular characterization of IncFII plasmid carrying bla_NDM-5_ in a Salmonella enterica serovar Typhimurium ST34 clinical isolate in China. mSphere 8, e0048023. doi: 10.1128/msphere.00480-23. PMID: 37909767 PMC10732066

[B129] Zeng Z. Lei L. Li L. Hua S. Li W. Zhang L. . (2022). In silico characterization of bla_NDM_-harboring plasmids in Klebsiella pneumoniae. Front. Microbiol. 13, 1008905. doi: 10.3389/fmicb.2022.1008905. PMID: 36504778 PMC9727287

[B130] Zhai R. Fu B. Shi X. Sun C. Liu Z. Wang S. . (2020). Contaminated in-house environment contributes to the persistence and transmission of NDM-producing bacteria in a Chinese poultry farm. Environ. Int. 139, 105715. doi: 10.1016/j.envint.2020.105715. PMID: 32315891

[B131] Zhang R. Li Y. Chen J. Liu C. Sun Q. Shu L. . (2023). Population genomic analysis reveals the emergence of high-risk carbapenem-resistant Escherichia coli among ICU patients in China. J. Infect. 86, 316–328. doi: 10.1016/j.jinf.2023.02.004. PMID: 36764393

[B132] Zhang F. Liu X. Li Z. Li Z. Lei Z. Fan Y. . (2025). Tracking international and regional dissemination of the KPC/NDM co-producing Klebsiella pneumoniae. Nat. Commun. 16, 5574. doi: 10.1038/s41467-025-60765-7. PMID: 40593598 PMC12216068

[B133] Zhang R. Liu L. Zhou H. Chan E. W. Li J. Fang Y. . (2017). Nationwide surveillance of clinical carbapenem-resistant Enterobacteriaceae (CRE) strains in China. EBioMedicine 19, 98–106. doi: 10.1016/j.ebiom.2017.04.032. PMID: 28479289 PMC5440625

[B134] Zhang S. Wang Y. Song H. Lu J. Yuan Z. Guo J. (2019). Copper nanoparticles and copper ions promote horizontal transfer of plasmid-mediated multi-antibiotic resistance genes across bacterial genera. Environ. Int. 129, 478–487. doi: 10.1016/j.envint.2019.05.054. PMID: 31158594

[B135] Zhang K. Xin R. Zhao Z. Li W. Wang Y. Wang Q. . (2021). Mobile genetic elements are the major driver of high antibiotic resistance genes abundance in the upper reaches of Huaihe River Basin. J. Hazard. Mater. 401, 123271. doi: 10.1016/j.jhazmat.2020.123271. PMID: 32629348

[B136] Zhao Q. Berglund B. Zou H. Zhou Z. Xia H. Zhao L. . (2021). Dissemination of bla_NDM-5_ via IncX3 plasmids in carbapenem-resistant Enterobacteriaceae among humans and in the environment in an intensive vegetable cultivation area in eastern China. Environ. pollut. 273, 116370. doi: 10.26855/jhass.2025.01.007 33460870

[B137] Zhao Y. A. Huang J. S. Shi Y. Ding H. Zhao Z. G. (2021). Molecular epidemiology of NDM genes in 23 carbapenem-resistant Escherichia coli isolates. Chin. J. Nosocomiology 31. doi: 10.3389/fphar.2025.1591724. PMID: 40606623 PMC12213433

[B138] Zou H. Jia X. Liu H. Li S. Wu X. Huang S. (2020). Emergence of NDM-5-producing Escherichia coli in a teaching hospital in Chongqing, China: IncF-type plasmids may contribute to the prevalence of bla_NDM-5_. Front. Microbiol. 11. doi: 10.3389/fmicb.2020.00334. PMID: 32210935 PMC7069339

[B139] Zuo H. Sugawara Y. Kondo K. Kayama S. Kawakami S. Uechi K. . (2024). Emergence of an IncX3 plasmid co-harbouring the carbapenemase genes bla_NDM-5_ and bla_OXA-181_. JAC-Antimicrob. Resist. 6, dlae073. doi: 10.1093/jacamr/dlae073. PMID: 38741895 PMC11089413

